# Colonization of weakened trees by mass-attacking bark beetles: no penalty for pioneers, scattered initial distributions and final regular patterns

**DOI:** 10.1098/rsos.170454

**Published:** 2018-01-03

**Authors:** Etienne Toffin, Edith Gabriel, Marceau Louis, Jean-Louis Deneubourg, Jean-Claude Grégoire

**Affiliations:** 1 Chimie Physique et Biologie Théorique, Université libre de Bruxelles, CP 231, boulevard du Triomphe, 1050 Bruxelles, Belgium; 2 LMA EA2151, Université d'Avignon, 84000 Avignon, France; 3 INRA - Unité BioSP, 84000 Avignon, France; 4 Spatial Epidemiology Lab (SpELL), Université libre de Bruxelles, CP 160/12, 50 av. FD Roosevelt, 1050 Bruxelles, Belgium

**Keywords:** collective foraging, resource partitioning, competition, bark beetles, public information, aggregation

## Abstract

Bark beetles use aggregation pheromones to promote group foraging, thus increasing the chances of an individual to find a host and, when relevant, to overwhelm the defences of healthy trees. When a male beetle finds a suitable host, it releases pheromones that attract potential mates as well as other ‘spying’ males, which result in aggregations on the new host. To date, most studies have been concerned with the use of aggregation pheromones by bark beetles to overcome the defences of living, well-protected trees. How insects behave when facing undefended or poorly defended hosts remains largely unknown. The spatio-temporal pattern of resource colonization by the European eight-toothed spruce bark beetle, *Ips typographus*, was quantified when weakly defended hosts (fallen trees) were attacked. In many of the replicates, colonization began with the insects rapidly scattering over the available surface and then randomly filling the gaps until a regular distribution was established, which resulted in a constant decrease in nearest-neighbour distances to a minimum below which attacks were not initiated. The scattered distribution of the first attacks suggested that the trees were only weakly defended. A minimal theoretical distance of 2.5 cm to the earlier settlers (corresponding to a density of 3.13 attacks dm^−2^) was calculated, but the attack density always remained lower, between 0.4 and 1.2 holes dm^−2^, according to our observations.

## 1. Background

Conspecific, but not necessarily related, animals can forage collectively [1,2] using available information produced by successful individuals [3]. While this behaviour increases the individual probability of success, it also raises largely unresolved questions regarding the benefits and penalties of cooperation. Once resources have been located, their partitioning could be equitable (which might lead to scramble competition at high densities) or biased to the advantage of some colonizers, resulting in the eviction of some candidate settlers. Bark beetles (Coleoptera, Curculionidae and Scolytinae) provide multiple examples of such strategies and the associated dilemmas. Many species forage collectively for the scarce, unpredictable and ephemeral resources of weakened or dead trees [4–9]. At high population densities, some even mass-attack and kill healthy trees [4,10–12]. In both cases, ‘pioneer’ individuals of either sex, depending on the species, find a host, initiate a gallery and simultaneously emit aggregation pheromones that attract conspecifics of both sexes [4,12,13]. This collective behaviour relies on high numbers of insects being in the air to discover a host and, if relevant, overcome its defences. Aggregation can also have other consequences, such as outcompeting pathogenic fungi [14] or diluting the impact of natural enemies [15].

In all cases, attack densities above a certain limit negatively impact fitness [11,12,14,16–22]. Density-regulating mechanisms thus appear necessary for both mass-foraging and mass-attack. Byers [23–25] suggested that *Ips typographus*, *Ips paraconfusus* Lanier, *Tomicus piniperda* (L.) and *Pityogenes chalcographus* (L.) on fallen trees (FTs) and *Dendroctonus brevicomis* Le Conte on healthy trees (HTs) solve or minimize density issues by regularly spacing new attacks on their hosts. Hedden & Gara [26] and Safranyik & Vithayasai [27] also recorded regular distributions of attack holes on *Dendroctonus pseudotsugae* (HTs) and *Dendroctonus ponderosae* (HTs), respectively, and Byers [23] used computer simulations of the attack holes by *I. typographus* on 1-m-long logs to estimate a 2.5 cm *minimum allowed distance* (MAD) to the nearest neighbour*.* Interestingly, this MAD remained approximately constant at the three attack densities (0.90, 1.99 and 3.17 holes dm^−2^) observed in the field that were used for calibrating the simulations, suggesting a fixed value for a given population under different circumstances. Byers [23] also remarked that the uniform nature of the natural attacks became more apparent at the highest density, i.e. as the mean distance to the nearest neighbour approached the MAD.

However, most studies have been centred on ‘tree-killing’ species in the act of overwhelming the defences of living, well-protected hosts (e.g. [11,28–30]), with relatively little attention being paid to the constraints and opportunities related to collective establishment on defenceless hosts (but see [6,11,14,23]). The collective and individual strengths and weaknesses of these strategies have been discussed from various perspectives ranging from implicit cooperation to outright competition between ‘pioneers’ and ‘followers’ that might turn into ‘cheaters’ [12,31].

When a notorious tree killer, the mountain pine beetle, *D. ponderosae* Hopkins, attacks a new host, the pioneer beetles first aggregate locally on the trunk, and the subsequent attack pattern on the same host gradually shifts towards a regular distribution [11]. The initial concentration on the host is interpreted as a collective weakening of the local tree defences, and the fact that this behaviour begins with a very local collective pressure on the attacked trees is illustrated by the occurrence of ‘strip- or patch-killed’ trees in which only local portions of the phloem have been colonized [11].

Up to a certain threshold, a higher attack density on a living tree is mutually beneficial for the pioneers and followers alike because it allows the local defences of a tree to be rapidly overcome [11,28,29]; therefore, aggregating at high densities on weakened trees apparently provides no collective gain. If landing en masse on a defenceless host is mainly or uniquely the consequence of group foraging, we can expect that insects adopt different strategies than concentrating on local tree defences. The objective of this study is to analyse the colonization patterns of a facultative tree killer infesting poorly defended hosts. Our main hypothesis is that the absence of consistent defences would influence the spatio-temporal deployment of the attacking beetles. A secondary question is whether, under these conditions, the status (risks and benefits) of the ‘pioneers’ should be reconsidered. We addressed these issues by monitoring the establishment of the European eight-toothed spruce bark beetle, *I. typographus* L., on weakened trees that were artificially or naturally felled.

## 2. Material and methods

### 2.1. Main observations on tree segments with pheromone lures

This study was initiated on 4 June 2013 and made use of a set of trees that had been uprooted by being pulled down at various times in the year and protected by screens so that they could be offered for colonization in a controlled fashion (see [7] for a complete description). The experimental protocol was primarily designed to answer other questions, i.e. the temporal changes in the nutritional value of windthrows for bark beetles [7] and the temporal changes in windthrow defences [8]. This latter study showed that the trees lost most of their defences with approximately 12 µg g^−1^ (dry weight) and 18 µg g^−1^ of the constitutive and induced terpenes, respectively (versus 2 mg g^−1^ (constitutive) and up to 18 mg g^−1^ (induced) in living spruces).

In addition, this set-up allowed the colonization kinetics to be quantified and the spatial pattern of attack holes to be characterized through time (the age of uprooted trees did not influence the results described in this work; electronic supplementary material, appendix S1). For the purpose of this study, each of the boles was divided into two observation segments (electronic supplementary material, figure S1): a 3-m-long basal segment starting from the tree collar and a 5-m-long upper segment starting 4 m from the collar.

The 1-m-long segment between the basal and upper segments was covered with a fine (0.4 mm) transparent PVC sheet to prevent colonization, and on four of the eight trees, insects slipping on this non-adhesive surface were collected in containers at the bottom of the bolts to estimate overall daily landing rates during the entire observation period. According to direct observations, approximately 70% of the landing insects were caught in the containers. The collected insects were sexed by genitalia extraction, and the sex ratio (SR = no. of males/no. of landed) remained stable throughout the observations (weighted linear relationship: SR = *f*(*t*), *F*_1,14_ = 0.098, *p* = 0.76) with a mean weighted value of SR = 0.38 (with weight corresponding to the number of sexed landed insects per day). The daily landing rate of males was computed from the daily landing rates using this mean SR.

In addition, because the two segments of each tree were physically separated by this mid-section, which was impassable by walking and whose length (1 m) was far greater than the MAD between attack holes (approx. 2.5 cm) as described by Byers [23], the lower and upper segments were each treated as independent replicates.

New entrance holes were marked with numbered pins, and the date of each attack was recorded for analysis. New holes were monitored at least daily until the 17th day (from 6 June 2013 to 23 June 2013) and approximately every 7 days thereafter (from 23 June 2013 to 9 July 2013, i.e. days 17–45). The lower part of the stem was not easily accessed as it was often close to, or touching, the ground. Therefore, we recorded what we could observe, and every record was included in the study. However, there were very few attacks on the underside.

To initiate colonization, two pheromone dispensers were applied per tree (sealed polythene bags containing 1.64 g of 2-methyl-3-buten-2-ol (MB) (Sigma–Aldrich, 98% chemical purity) and 0.08 g of (*S*)-*cis*-verbenol (cV) (Sigma–Aldrich, 95% chemical purity; P50% optical purity) [32] on each segment (2.5 m, basal segment; 5.5 m, upper segment)). Release rates were 17 mg d^−1^ and 2.6 mg d^−1^ for MB and cV, respectively, as estimated from the weight loss in a wind tunnel (0.06 m s^−1^, 20°C). Pheromone dispensers were removed when 20 entrance holes were counted anywhere around the trunks within 50 cm of both sides of the dispensers (electronic supplementary material, figure S1).

The pheromones were removed from 12 segments before the end of the observations after a median exposition time of 100 h [78; 148.5] (*N* = 13), corresponding to 61.3 ± 16.1% of the total number of entrance holes (*H*_END_) counted eventually. One segment retained its pheromone dispenser and displayed 0.57 attacks dm^−2^. Statistical analysis showed that the pheromone dispensers did not influence the spatial colonization pattern (deviation test: basal segment, *p* = 0.355; upper segment, *p* = 0.366; see electronic supplementary material, appendix S3).

Three of the 16 segments exhibited particularly low numbers (<30 holes) despite having colonization dynamics similar to those of the other segments, and significant bark decay was observed on two of them. Thus, these three segments were discarded from our analysis, which ultimately considered a total of 13 segments.

### 2.2. Validation: observations of naturally infested, whole trees

This part of the study focused on three spruces in Smuid (Province of Luxembourg, Belgium) that were windfelled in the winter of 2014–2015 and remained on the ground. Roots were partially in contact with the soil, and the trees were pruned up to, but excluded, the crown before the observations began. No pheromone dispenser was added, and colonization occurred spontaneously over the trees. New entrance holes were marked with numbered pins every 1–3 days, depending on the weather conditions, during the main flight period from 5 June 2015 to 8 July 2015.

### 2.3. Data collection

Once the observations were complete, the segments or the attacked parts of the whole trees were wrapped in cellophane sheets, on which the position and number of the pins were marked. The sheets were then removed and photographed in the laboratory, and the successive pictures were stitched together prior to analysis. The Cartesian coordinates of the attack holes were measured using ImageJ software (v. 1.49t) [33] with the *X*-axis corresponding to the positions along the length of the tree, while the *Y*-axis corresponded to the positions around the circumference of the tree with *y* = 0 corresponding to the upper rim of the tree.

In addition to the entrance holes, patches of bark showing a rough texture (mostly located around branches while the other parts of the bark usually showed a smooth surface; electronic supplementary material, figure S2) were delineated on the cellophane sheet. The area of each type of bark (rough and smooth) and the repartition of the holes among these two categories were determined using ImageJ software. These data were not available for two segments (tree 8, segments 15 and 16).

### 2.4. Statistical analysis

When the normality of the distributions was confirmed (Shapiro–Wilk test), the results were presented as the mean ± s.d. (*N*); otherwise, medians [Q1; Q3] (*N*) were used. The significance level for all statistical tests was *α* = 0.05.

#### 2.4.1. Colonization dynamics

Colonization dynamics were analysed by considering the entire surface available on each segment of area, *A*_segment_ (in dm^2^), as a proxy of its maximum carrying capacity, and the number of attack holes, *H*, at each time step. The associated attack density, hereafter referred to as segment density, was defined as *Λ*_segment_ *=* *H*/*A*_segment_ (holes dm^−2^).

#### 2.4.2. Spatial patterns

The R [34] package spatstat [35] was used to analyse the spatial pattern of the attack holes and its departure from *complete spatial randomness* (CSR, which is synonymous to the homogeneous Poisson point processes [36]). Spatial pattern analyses were conducted after computing the region of interest defined by the convex hull of the infested patch, i.e. the most compact convex polygon encompassing all the entrance holes at the end of colonization (figure 3*a*) whose area was defined as *A*_hull_ (dm^2^). The attack density on the convex hull, hereafter referred to as hull density (λ_hull_), was computed at different times during colonization as λ_hull_ *=* *H*/*A*_hull_ (holes dm^−2^).

#### 2.4.3. Pointwise envelopes

Departure from CSR was assessed by comparing the *G*-function (nearest distance between two attack holes) and the *L*-function (Besag's transformation of Ripley's *K*-function, i.e. the number of events within a given distance from any particular event), which were calculated from the observed patterns to an envelope of Monte Carlo simulations of homogeneous Poisson processes [36,37] within the convex hulls. A spatial pattern is considered regular if its related *G*-function (or *L-*function) is located below the lower Poisson envelope at some distances and clustered if located above the upper Poisson envelope (for the interpretation of confidence envelopes, see [38]). The transformed distribution *G′*(*r*) can be used to stabilize the variance of *G*(*r*): $G^{\prime }(r)=\arcsin(\sqrt{G(r)})$ [37].

#### 2.4.4. Diggle–Cressie–Loosmore–Ford test

The Diggle–Cressie–Loosmore–Ford (dclf) test performs hypothesis tests for goodness of fit of a point pattern dataset to a point process model, based on Monte Carlo simulation from the model. It is a test based on a statistic, $U=\int_{0}^{r_{\max}}{\left(T(r)-\bar{T(r)}\right)}^{2}\;\text{d}r$, where *T* is a summary function (*G′*(*r*), *L*(*r*) functions) and *r*_max_ is a chosen upper limit distance over which the maximum absolute deviation will be computed for the test (Baddeley *et al*. [37] suggest that it should be slightly larger than the maximum possible range of interactions between points). The function *T*(*r*) is calculated from the observed data, *T*_obs_(*r*), and from *N* simulated point patterns under a null hypothesis, *T_i_*(*r*), *i* *=* 1, *… *,*N*, and its average is denoted as $\bar{T(r)}$. We used the *G′*- and *L*-functions as summary statistics to test for significant departures from CSR. Agreement between the statistical analyses of both functions was necessary to characterize any given pattern as regular or random (CSR).

Using the same functions and methods, the density of regular pattern detection, λ_detection_ (i.e. the density at which a regular pattern is discriminated from CSR), was determined for each segment by iteratively computing the dclf test *p*-value of the successive steps of the colonization patterns.

We characterized the global spatial pattern exhibited among all the segments by aggregating the statistical results for each replicate. The global nearest-neighbour distance distribution function from those evaluated for each replicate, *G_i_*(*r*), *i* *=* 1, … ,*n*_rep_, is defined as follows:

2.1 $$G(r)=\overset{n_{\mathrm{rep}}}{\underset{i=1}{\sum }}G_{i}(r)\frac{n_{i,r}}{n_{r}},$$

where *n*_rep_ is the number of replicates; *n_i_*,*_r_* is the number of points in the eroded region obtained by trimming off a margin of width *r* and $n_{r}=\sum_{i=1}^{n_{\mathrm{rep}}}n_{i,r}$.

#### 2.4.5. Simulating random colonization with an inhibition distance

The mechanisms impeding additional holes within a certain distance of earlier attacks remain unknown, but they could pertain to inhibition or to a lack of stimulation. In this study, however, we will label these unknown mechanisms as ‘inhibition’ for the sake of concision. Random colonization with an inhibition distance *r* (cm) was simulated using a *simple sequential inhibition* (SSI) [36,38] algorithm provided by the rSSI function of the R package spatstat as follows. At each iteration, a new entrance hole was generated at random within the environment and independently of the preceding holes. If the new hole was closer than the distance *r* to any pre-existing attack, it was rejected, and another random entrance hole was generated. Under default settings, the algorithm was terminated when the *N* holes were placed on the area.

The SSI algorithm uses an inhibition distance *r* defining a circular rejection area. This agrees well with preliminary investigations, indicating that the distributions of nearest-neighbour distances along the *X*-axis (4.22 cm [2.1; 6.51] (*N* = 1614)) and *Y*-axis (4.21 cm [2.37;6.23] (*N* = 1614)) are similar (Wilcoxon rank sum test: *W* = 1 286 935, *p* = 0.56).

## 3. Results

### 3.1. Colonization dynamics of the tree segments

**Figure 1.**
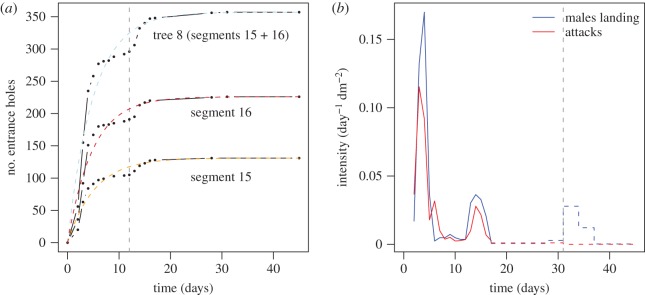
Infestation dynamics. (*a*) Typical colonization dynamics, here on tree 8 and its segments (basal no. 15 and upper no. 16). Black dots, observed points; dashed lines, nonlinear fit (blue, entire tree 8; orange, segment 15; red, segment 16); vertical grey dashed line, beginning of the second colonization stage. (*b*) The time series of daily male landing intensity (sum of the four traps) and the daily appearance of new entrance holes on the segments (sum of the 13 segments) are well correlated. Daily intensities are computed as intensity_Land_ = (no. landing per day × SR)/area traps and intensity_Hole_ = no. new holes per day/total segments area. The grey vertical dashed line indicates the 31st day of the experiment, when a third landing wave (or at least partly a wave of re-emerging beetles caught in the landing traps) was observed without any additional entrance holes on the segments. The dashed red and blue lines indicate mean values computed from an intercensus duration greater than 1 day.

The temporal colonization dynamics of the segments was nonlinear: the colonization rate rose for a short period and then constantly decreased until the number of entrance holes reached a plateau *H*_END_ = 124.31 ± 43.18 (*N* = 13) (figure 1*a*). The proportion of entrance holes visible at first measurement (day 2) was *H*_1_/*H*_END_ = 0.12 [0.09; 0.19] (*N* = 13). Fifty per cent of the final number of holes occurred within the first 4 days [4; 4] (*N* = 13), and 90% (all segments combined) were observed at day 14.

These colonization dynamics can be fitted to the following function:

3.1 $$H(t)=H_{\mathrm{END}}(1-{\text{e}}^{-\beta t}),$$

where *H*_END_ stands for the number of entrance holes counted at the end of the observations and *β* controls the steepness of the curve with high *β* values leading to shorter durations to reach *H*_END_ (nonlinear fitting: *β* = −0.206 ± 0.07 d^−1^, *N* = 13, with *R*^2^ ranging 0.92–0.99). There was no relationship between *β* and *H*_END_ (linear regression, *p* = 0.859). The colonization dynamics at the tree scale (i.e. with *H*(*t*) being the sum of the attacks on each tree) showed similar profile and fitting curves (nonlinear fitting: *β* = −0.204 ± 0.06 d^−1^, *H*_END_ = 230.9 ± 78.9, *N* = 7, with *R*^2^ ranging 0.93–0.98).

Two successive stages can be observed on almost every segment: a first stage from day 0 (initiation of observations) to day 11, during which the number of entrance holes reached a quasi-plateau (*H*_FIRST_), followed by a second stage from day 12 to day 45 (end of observations) leading to a second plateau (*H*_SECOND_ with *H*_FIRST_ *+* *H*_SECOND_ *=* *H*_END_). However, most of the attacks occurred during the first stage (*H*_SECOND_/*H*_FIRST_ = 0.311 [0.207; 0.379], *N* = 13). There was no relationship between the values of *H*_FIRST_ and *H*_SECOND_ (electronic supplementary material, figure S3).

The daily colonization intensity was well correlated to the number of males landing during all the observations (figure 1*b*; Pearson's product–moment correlation, *ρ* = 0.91, *t* = 9.34, d.f. = 19, *p* < 0.0001), which indicates that the observed colonization dynamics are directly influenced by insect availability; the increase in the number of landing males from the first to the second measurement probably explains the short increase in the rate of attack observed at the beginning of colonization (figure 1*a*). In addition, the landing rate strongly correlates with the average temperature measured during the observations (electronic supplementary material, appendix S7 and figure S4), which is known to influence the emergence and flight behaviour of the beetles. These results indicate that the two plateaus observed in the global shape of the colonization dynamics only result from a beetle shortage, but the stagnation of the number of entrance holes, despite the observation of a third landing stage on the 31st day (figure 1*b*), suggests that other regulating mechanisms may be at work, or could reflect at least partly the activity of re-emerging beetles caught in the landing traps while trying to leave the trees.

The area of the segments (*A*_segment_) showed a linear relationship with the area of the convex hull (*A*_hull_; *A*_hull_ = 0.72 × *A*_segment _− 18.8 dm^2^, *R*^2^ = 0.73, *F*_1,11_ = 33.8, *p* < 0.0001), while the final number of entrance holes was not related with the area of the segments (*H*_END_ = 0.21 × *A*_segment_ + 64.9, *R*^2^ = 0.03, *F*_1,11_ = 1.36, *p* = 0.27). This means that the probability of colonization per unit area is not equal between segments. By contrast, the final numbers of holes and the areas of the convex hulls were related (*H*_END_ = 0.46 × *A*_hull_ + 39.7, *R*^2^ = 0.30, *F*_1,11_ = 6.19, *p* = 0.03), which agreed with the complementary analysis (electronic supplementary material, appendix S8) and indicated that for density values similar to those observed in our observations, the number of holes influences the area of the convex hull in a predictable fashion. The following analysis indicated that the different trees are characterized by different intrinsic receptivities that can explain these differences.

To investigate the high variability in both the first plateau (*H*_FIRST_) and the final number of holes (*H*_END_) on each segment, we simulated the installation of 1614 entrance holes (the total number of entrance holes on the 13 segments) within 13 compartments with areas similar to those of the segments, using an SSI with *r* = 0 (with this null inhibition distance, the infestation followed a random Poisson process). The comparison of the observed densities at the end of the colonization process with those obtained by 10 000 simulations indicated that colonization was not random among segments (two-sample Kolmogorov–Smirnov test, *D* = 0.364, *p* < 0.032), which suggests that the variability in the plateau value *H*_END_ between observations was due to either a heterogeneous distribution of the flying insects around the segments and/or a difference in susceptibility between the segments.

Therefore, we describe the colonization of the segments as follows:

3.2 $$\frac{\text{d}H_{i}}{\text{d}t}=\alpha_{i}A_{i}\Phi (t)$$

and

3.3 $$H_{i}=\alpha_{i}A_{i}\int_{0}^{T}\Phi (t)\;dt,$$

where *Φ*(*t*) is the instantaneous flow of flying insects at time *t*; *A_i_* is the area of the segment *i*; *α_i_* is the susceptibility of this segment; and *H_i_* is the cumulative number of entrance holes on this segment at time *t*.

Coupling the equation (3.3), the relative colonization of each segment compared with the others is described as follows:

3.4 $$\frac{H_{i}}{\alpha_{i}A_{i}}=\frac{H_{j}}{\alpha_{j}A_{j}},$$

where the density *Λ_i_* of segment *i* is *Λ_i_* = *H_i_*/*A_i_*. The homogeneous flow of flying insects, *Φ*(*t*), around the segments is indicated by a linear relationship between the segment densities, *Λ_i_* and *Λ_j_*, with a slope defined as the ratio of the susceptibility of each segment, *α_i_*/*α_j_*.

Each pair of segments shows a characteristic relationship between their *Λ* values (for a plot of all relationships among pairs, see electronic supplementary material, figure S6). During the first colonization stage, a linear relationship describes the link between the *Λ* of each couple at any time well (*R*^2^ = 0.97 [0.95; 0.99], *N* = 78), indicating that the density of flying insects is similar around each segment and that there is no amplification or competition between them. When the second colonization stage occurs, the relationship remains linear, but the value of the slope is significantly different from that in the first stage in 70% of the pairs (using segment 1 as a reference with susceptibility *α*_1_ = 1; first stage: *α*_FIRST_ = 1.42 ± 0.57, *N* = 13; second stage: *α*_SECOND_ = 2.73 ± 1.78, *N* = 13). There was no significant relationship between both susceptibility values (*α*_FIRST_ and *α*_SECOND_) in any given segment. Moreover, the variation in the susceptibility of the segments (Δ*α* = *α*_SECOND _− *α*_FIRST_) was not related to the density at the end of the first colonization stage (*Λ*_FIRST_), which indicates that the number of entrance holes that occurred during the first stage did not influence, in a predictable fashion, the number of additional holes occurring during the second stage. There was a strong relationship between the initial receptivities (*α*_FIRST_) of the basal and upper segments of any given tree (*α*_FIRSTupper_ = 1.25 × *α*_FIRSTbasal_ − 0.05, *R^2^* = 0.81, *F*_1,4_ = 22.5, *p* = 0.009), indicating that each tree was characterized by its own intrinsic susceptibility.

### 3.2. The location of the entrance holes is strongly related to the texture of the bark

**Figure 2.**
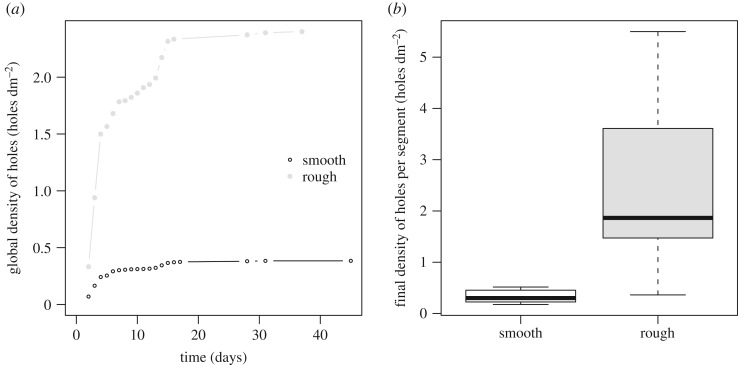
Rough bark exhibits a higher entrance hole density. (*a*) The global (all segments regrouped) density of holes is far higher on rough bark than on smooth bark during the entire experiment (global final hole density: *Λ*_smooth_ = 0.37 holes dm^−2^; *Λ*_rough_ = 2.33 holes dm^−2^). (*b*) At the end of the experiment, each segment shows a far greater hole density on rough bark than on smooth bark (per segment: *Λ*_smooth_ = 0.30 holes dm^−2^ [0.23; 0.46], *N*** **= 11; *Λ*_rough_ = 1.86 holes dm^−2^ [1.47; 3.61], *N*** **= 11; Wilcoxon matched-pairs signed-ranks test: *U* = 1, *p* < 0.001). Similar results were obtained when comparing density of attacks on smooth and rough barks on each entire tree (density of attacks on all rough bark on a tree versus density of attacks on all rough bark on a tree; Wilcoxon rank sum test: *W* = 0, *p* = 0.031).

The global density of entrance holes (all segments regrouped) at the end of the observations was 6.3 times higher on rough bark (final *Λ*_smooth_ = 0.37 holes dm^−2^; *Λ*_rough_ = 2.33 holes dm^−2^; figure 2*a*). Moreover, the hole density on rough bark was linearly related to that on smooth bark at the same time step (*Λ*_rough_ = 6.55 × *Λ*_smooth_−0.001, *F*_1,13_ = 1.35 × 10^4^, *p* < 0.0001, *R*^2^ = 0.99), indicating that the respective rates of discovery and infestation of both textures remained constant throughout the observations. This higher density of entrance holes on rough bark was observed on each segment (figure 2*b*). We did not find any relationship between the area of each texture or the ratio of rough bark with either plateau value of hole number (*H*_FIRST_ and *H*_SECOND_) (linear regression: *p* > 0.05).

### 3.3. The spatial pattern of entrance holes involves a spacing mechanism

#### 3.3.1. The final spatial pattern of entrance holes is regular

**Figure 3.**
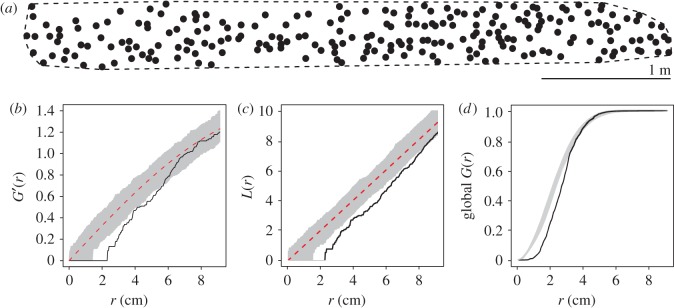
Statistical pattern analysis of segment 16 at the end of experiment. (*a*) Pattern of entrance holes circumscribed by the convex hull of the segment (density of attacks λ_hull_ = 0.730 holes dm^−2^). (*b*) Pointwise envelope of the *G′*-function and (*c*) *L*-function. Black line, function of the observed pattern; red dotted line, function of the theoretical CSR pattern of density λ; grey shading, pointwise envelopes of 999 simulated CSR processes of density λ. Significant departures from CSR indicating a regular pattern are observed for both functions (experimental line below the lower envelope: *G′*-function: dclf test *U* = 0.30, rank = 1, *p* = 0.001; *L-*function: dclf test *U* = 16.88, rank = 1, *p* = 0.001). (*d*) Global nearest-neighbour distance distribution *G*(*r*) obtained from the experimental data (all replicates regrouped; black line) and the confidence envelope obtained from the *N* = 999 simulated Poisson point processes (grey shading).

The pointwise envelopes (figure 3*b–d*) and dclf tests indicated that nine of the 13 segments showed a regular attack distribution at the end of the observations (dclf test: *p* ≤ 0.05), while the other four were characterized by a random pattern (dclf test: *p* > 0.05 for one of the *G′*- or *L-*based dclf tests; electronic supplementary material, table S2). It is worth noting that the distribution of the entrance holes on three of these four segments, all of which were characterized by low density values, was close to a regular pattern (*p* ≤ 0.05 for one of the *G′*- or *L-*based dclf tests, with *p* close to 0.05 for the other test; electronic supplementary material, table S2). This suggests that, had colonization continued, the pattern on these four segments would have been characterized as regular.

Moreover, a statistical analysis considering all replicates together indicated that the data were more regular than under a CSR hypothesis (figure 3*d*) because the observed curve is under the CSR envelopes for short distances. This result was confirmed by the dclf test with *p* = 0.001. In this test, the integral deviation measure obtained from the data was $U_{\mathrm{obs}}=\int_{0}^{r_{\max}}{\left(G(r)-\bar{G(r)}\right)}^{2}\;\text{d}r$, with *r*_max_ = 20 cm and $\bar{G(r)}$ as the mean of *G*_1_(*r*),* *… ,*G_N_*(*r*), as defined in equation (2.1), and evaluated based on the *N* = 999 independent Poisson process simulations.

#### 3.3.2. Regularity arises when a sufficient entrance hole density is achieved

**Figure 4.**
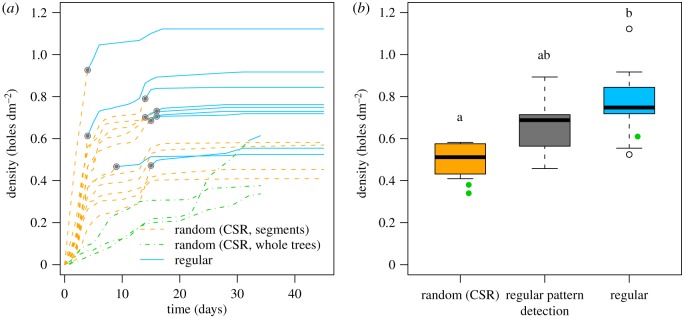
(*a*) The density of entrance holes increases monotonically during colonization. For both segments and whole trees, entrance hole patterns are discriminated from CSR (segments, orange dashed line; whole trees, green dashed line) and characterized as regular (blue solid line) as the density of the holes increases. Grey dots indicate the density of regular pattern detection λ_detection_. (*b*) Hole density λ_hull_ (holes dm^−2^) on each segment as a function of the exhibited pattern (orange and blue boxes, final density recorded at the end of segment observation; grey box, density λ_detection_ recorded during regular pattern detection). Random (CSR): 0.51 [0.44; 0.57], *N*** **= 4; regular pattern detection: 0.69 [0.56; 0.71], *N*** **= 9; regular: 0.75 [0.72; 0.84], *N*** **= 9. Letters indicate significant differences indicated by the Kruskal–Wallis *post hoc* test. Green dots stand for the density recorded at the end of the whole trees experiment.

While entrance hole patterns appeared to be randomly distributed at low densities (CSR), those observed at high densities were regular (electronic supplementary material, table S2), suggesting the existence of a minimal density at which a regular pattern can be discriminated from CSR (λ_detection_ = 0.69 attacks dm^−2^ [0.56; 0.71], *N* = 9; figure 4*b*; Kruskal–Wallis $\chi_{2}^{2}=6.637$, *p* = 0.036; *post hoc* test: the only significant difference was between final densities of random and regular patterns; electronic supplementary material, table S3), with newcomers filling the empty spaces (this will be confirmed later). The density allowing the detection of a regular pattern was almost twice as high as the density recorded at the time of pheromone lure removal (λ_detection_/λ_pherom_ = 1.50 [1.16; 1.67], *N* = 9).

#### 3.3.3. The distance to the nearest neighbour decreases with hole density

**Figure 5.**
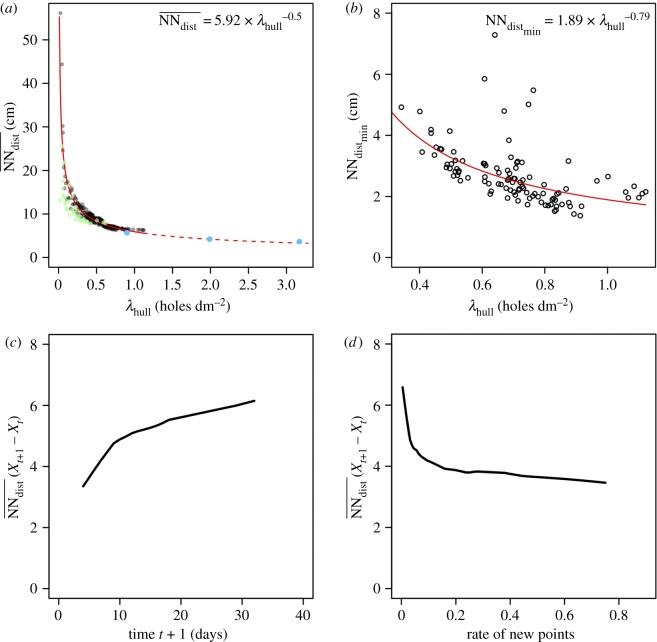
(*a*) The mean nearest-neighbour distance ($\bar{\text{N}{\text{N}}_{\mathrm{dist}}}$) decreases monotonically with hull hole density (λ_hull_) from the beginning to the end of the experiment. The mean fit obtained by pooling values of all segment replicates and its predictions for λ_hull_ > 1 (dashed red curve) are in good agreement with the values observed for whole trees (green dots) and those of Byers [23] (blue dots). (*b*) The minimum nearest-neighbour distance observed at each time step $\left({\text{NN}}_{{\mathrm{dist}}_{\min}}\right)$ decreases with hull density (λ_hull_), and this trend fits a power law curve well $\left({\text{NN}}_{{\mathrm{dist}}_{\min}}=1.97\times {\lambda_{\mathrm{hull}}}^{-0.78};\;r^{2}=0.30\right)$. (*c*) Mean distance to the nearest neighbour ($\bar{\text{N}{\text{N}}_{\mathrm{dist}}}$) between attacks at time *t* and new attacks at *t* + 1 with reference to time *t* + 1 (in days). (*d*) The same with reference to the rate of new points between times *t* and *t* + 1.

The mean distance to the nearest neighbour (NN_dist_) decreased monotonically with density following a power law of the form $\text{N}{\text{N}}_{\mathrm{dist}}=\gamma \times \lambda^{\alpha }$ (figure 5*a*). This statistically significant relationship was observed in each replicate (*γ* = 6.10 ± 0.39; *α* = −0.44 ± 0.11; *r^2^* = 0.96 ± 0.04, *N* = 13). Figure 5*a* shows the mean curve fitted to the values of all segments combined (*γ* = 5.92; *α* = −0.50; *r^2^* = 0.92).

#### 3.3.4. Variations in the spacing mechanism are partly explained by entrance hole density

The distance at which the nearest-neighbour distance function *G*(*r*) first crosses the lower CSR envelope indicates the existence of an inhibition mechanism regulating beetle establishment and provides a first approximation of the inhibition distance (MAD). Figure 5*b* shows that *I. typographus* iteratively fills the empty space using a spacing mechanism. Moreover, the minimum nearest-neighbour distance $\left({\text{NN}}_{{\mathrm{dist}}_{\min}}\right)$ observed at each time step decreases with time/holes density, giving a more accurate estimation of the inhibition distance as it tends towards its actual value as colonization proceeds.

### 3.4. Colonization is dynamic, homogeneous and non-diffusive

#### 3.4.1. Attacks fill the available space on the tree

**Figure 6.**
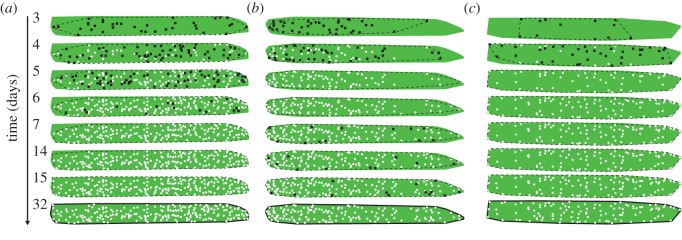
Characteristic spatio-temporal colonization patterns: (*a*) segment 16, (*b*) segment 8 and (*c*) segment 14. The entrance holes are initially distributed at random, and the remaining vacant area is gradually occupied (from top to bottom; absolute time in days indicated on the left side). Black squares represent entrance holes at the current time step, and white dots represent earlier holes. Dashed lines indicate convex hulls at each time step, while solid lines depict the final convex hulls used for the pattern analysis and computation of hull density **λ**_hull_ at each time step. This final convex hull is also indicated at each step by the green shading in the background.

The spatio-temporal pattern of attack holes was similar between replicates. The first entrance holes were randomly located, covering almost the entire segment (figure 6), and the following holes were scattered over the segment, filling the empty space. Figure 5*c*,*d* shows this phenomenon: the mean nearest-neighbour distance between attacks at times up to *t* and attacks occurring at the next time step, *t* + 1, tends to increase with time (figure 5*c*). However, because new attacks are more numerous at the beginning of the infestation (figure 1*a*), the inverse trend is observed when the rate of new attacks between time step *t* and *t* + 1 is considered: this relationship indicates that new entrance holes appear closer to each other. This empirical description was confirmed by a complementary analysis based on statistical methods dedicated to the study of point process patterns (electronic supplementary material, appendix S12) that indicated that there are interactions between the attacks (i.e. inhibition) throughout the entire colonization process. Moreover, it appeared that the sole filling of the empty areas by new entrance holes could explain the observed decrease in the nearest-neighbour distance (electronic supplementary material, appendix S13); the average and observed distance to the nearest neighbour decreases with increasing density (density increases with time). Additional analysis suggested that the inhibition distance is barely affected by the age of the attacks (electronic supplementary material, appendix S17).

#### 3.4.2. Colonization is homogeneous over the entire available surface

The spread of the entrance holes along the *X*-axis (distance between the most distant projected holes) at the first measurement was close to that measured at the end of the observations; the spreads of holes along the *X*-axis at the first measurement (spread_1_) and the final pattern (spread_END_) showed a strong linear relationship (spread_1_ = 0.782 × spread_END_ + 22.120, *F*_1,11_ = 25.5, p≪0.001, *R^2^* = 0.67; electronic supplementary material, figure S8*a*). These patches of entrance holes covered almost the entire available length (seg.length) of each segment (spread_END_/seg.length = 0.95 [0.91; 1.00]; *N* = 13). It should be noted again that the location of the pheromone dispenser had no influence on hole locations (for a formal test, see electronic supplementary material, appendix S3).

The fraction of all entrance holes mapped that were present at the first measurement (*H*_1_/*H*_END_) ranged from 4% to 48% (median = 15.3 [9.90; 19.0]; *N* = 13). The number of holes at this first measurement (*H*_1_) did not influence the spread of the holes over the *X*-axis. The number of holes quickly reached almost the maximum value (spread_1_/spread_END_ = 0.72 + 0.00356 × *H*_1_, *F*_1,11_ = 1.53, *p* = 0.24). In addition, the coverage of the colonized patch quickly approached its maximal value: on day 3, the relative coverage was 73.1 ± 11.9% (*N* = 13), while 35.7 ± 11.6% (*N* = 13) of the final number of holes was recorded.

Moreover, there was no relationship between the time and location of the entrance holes along the *X*-axis (electronic supplementary material, figure S8*b*) in nine of 13 replicates. In the four replicates showing a statistical relationship, this trend was very mild considering the low *R*^2^ values (maximum = 0.21) and the small displacement of the centre of gravity of the entrance holes, *X*, through time (maximum of 16.6 cm over 30 days on a 5-m-long segment; electronic supplementary material, table S4). This indicates that the segment was filled with entrance holes without any preferential location during the entire observation period.

In terms of spatial pattern, the spread of entrance holes over the segment length (at first measurement or at the end of the observations), the mean nearest-neighbour distances ($\bar{{\text{NN}}_{\mathrm{dist}}}$) and the densities of regular pattern detection (λ_detection_) seem unaffected by segment size (electronic supplementary material, table S5).

Finally, we conducted simulations of random attacks over segments with different receptivities under the sole regulatory mechanism of an inhibition distance (electronic supplementary material, appendix S16). This theoretical analysis indicated that no plateau occurred within the range of the observed entrance hole densities and at inhibition distances compatible with our results (electronic supplementary material, figure S9). Hence, the inhibition distance controls the location of successive holes over a small range (a few centimetres), but does not significantly influence the infestation rate at the global scale (metre scale) at common realistic densities.

### 3.5. Validation: observations on naturally infested whole trees

The three whole trees showed the same colonization dynamics and similar spread characteristics as the segments, confirming that the pheromones used in the observations described here, or the splitting of each tree into segments, did not influence the spatio-temporal patterns (figures 4*a*,*b* and 5*a*; electronic supplementary material, table S1).

## 4. Discussion

Our analyses show that colonization first occurs without any preferred location or direction; the insects apparently initially establish anywhere over almost the entire available surface. A regular pattern is then progressively achieved by randomly occupying the still-vacant areas, leading to a constant decrease in the distance to the nearest neighbour down to a minimum below which new entrance holes are not excavated. The theoretical values that we predicted agree well with those reported by Byers [23] (figure 5*a*, blue dots), but this minimum was rarely reached in our observations.

Interestingly, our description of the establishment of beetles on FTs, i.e. initially covering most of the available bark surface and eventually being regulated by a short-distance ‘inhibition’ mechanism, strongly contrasts with that of Raffa & Berryman [11], who reported that during the colonization of healthy standing pines by *D. ponderosae*, ‘the galleries were highly clustered at low attack densities. The degree of clustering then declined as the number of entries increased, approaching uniformity at high densities’. A final regular distribution in the attacks of *D. ponderosae* on standing trees has also been reported by Safranyik & Vithayasai [27]. By contrast, we observed that the first landings occurred anywhere on the trees. This, together with the findings of Louis *et al*. [8] that FTs are very weakly defended, supports our hypothesis that aggregation on FTs has no, or very little, relation to overcoming their resistance. Aggregation on a weak host could thus mainly be a by-product of mass-foraging (many beetles in the air needed to spot the resources), but other benefits (e.g. escaping from natural enemies or side-stepping competitors) should not be excluded. Although there is a possibility that spatial attack patterns differ between bark beetle species, the most likely possibility is that the constraints and mechanisms at play on weakened hosts radically differ from those governing the colonization of standing trees. Further measurements are needed to complete this comparison.

A striking aspect of our results is that many attacks occurred almost simultaneously. Within 5 days, 37.7–87.4% of the beetles established on the segments. According to Chararas [39] and our own unpublished observations, the egg galleries of *I. typographus* increase by approximately 4 mm d^−1^, roughly corresponding to the laying of one or two eggs. Therefore, the very early settlers not only risked very little but also gained very little leeway. On standing trees, being the first to attack represents a serious risk, and some evidence has been gathered in *D. ponderosae* that suggests that pioneering constitutes a ‘desperation’ strategy chosen by individuals that have already exhausted their resources during their quest for a host [40,41] and that followers on standing trees have higher reproductive success than the pioneers. As followers exploit a host found by others at some cost, they can be seen as ‘cheaters’ [12,31] although they also contribute to killing the host. On FTs, there is no such penalty for ‘pioneering’, as the first male beetle that lands is theoretically able to establish.

According to Stamps & Krishnan [42], new settlers benefit from larger territories when they establish while respecting a ‘neighbour rule’, i.e. a minimal distance from the neighbour. In this study, the new settlers appeared to establish at random in all respects, sometimes far from any neighbour but maintaining a minimal distance to their already-established neighbours.

Our simulations (electronic supplementary material, appendix S16) showed that a maximum density of 3.13 attacks dm^−2^ can occur at an ‘inhibition’ distance of 2.5 cm. This is consistent with the values observed in the field for felled (0.25–3.5 attacks dm^−2^ [22,43–46]) and standing trees (1–4 attacks dm^−2^ [19,44,46,47]). In our observation set-up, attack density always remained between approximately 0.4 and 1.2 holes dm^−2^ (figure 4*a*), suggesting that other regulatory mechanisms operate before this theoretical density is reached. As many landings occurred almost simultaneously, the information perceived by new potential settlers must have been conveyed very rapidly by the first beetles that established.

The mechanisms possibly regulating attack density have been thoroughly discussed by Byers [24], and they include avoiding landing in densely colonized areas or leaving these areas after landing, maintaining a sufficient distance from existing entrance holes above or within the bark and re-emerging when brood densities are too high. In our observations, it is notable that the last wave arriving on the trees did not establish at all (figure 1*b*) (with the reservation, however, that this last wave could have been, at least partly, formed by re-emergent beetles trying to leave the tree). Landing without attack has already been described by Anderbrant *et al.* [48], who reported a 15.8 : 1 ratio of beetles landing versus attacking, but a 3 : 1 ratio was reported by Paynter *et al.* [49]. The fate of the beetles that chose not to attack is unknown, but when provided with small bark slabs and spruce twigs, adult *I. typographus* has shown a relatively long survival capacity of more than 14 days [50] to more than 25 days [51]. A high dispersal capacity is also reported in this species [51], with up to 6 h and 20 min of flight in a flight mill and one-fourth of the tested beetles flying at least 1 h, corresponding to a distance of 18 km at an estimated speed of 5 m s^−1^. These capacities probably allow the insects to be fairly selective in terms of their breeding sites. In addition, *I. typographus* is probably flexible in its acceptance of a host, and its previous experience could influence further choices, as reported for *Ips pini* by Wallin & Raffa [52]. Under conditions of high population density, this could push the insects towards eventually accepting settlement at higher densities (that our simulations have shown as compatible with the 2.5 cm ‘inhibition’ distance; electronic supplementary material, appendix S16), or at even larger population densities, the insects could be led to shift to standing hosts [20].

Bark texture plays a strong role in the choice of attack locations. The rough bark at the base of the branches exhibits higher entrance hole densities than the smooth bark covering the rest of the segments. There is no indication that the colonization process could end due to the saturation of the preferred rough bark, and the ratio of densities on each bark type remains stable, suggesting that the discovery of rough bark is due to random, non-oriented walks on the hosts. This frequent settlement around branches is likely to be caused by irregularities in these tissues that favour the initiation of an entrance hole. Paynter *et al*. [49] report that *I. typographus* mostly explores crevices during host colonization, and similar observations have been reported for *D. ponderosae* by Safranyik & Vithayasai [27] and Ferrenberg & Mitton [53], and for *Ips calligraphus* by Haack *et al*. [54]. Such thigmotactic behaviour, which is observed in many other arthropods [55–58], could be sufficient to arrest the beetles close to branches and lead to their settlement on patches of rough bark. Moreover, a stable ratio of densities on each bark type and these behavioural observations are also consistent with the ideal free distribution theory (IFD) [59,60]. The IFD states that the density of individuals that will settle in various patches, e.g. patches of rough and smooth bark, is proportional to the amount of resources available in each. For bark beetles, the roughness that facilitates excavation is itself a resource but should also be an indicator of bark quality.

Although a number of unsolved issues should be explored further (spacing mechanisms, possible changes in fitness relative to the order of arrival, and the flexibility allowed to each individual to complete host selection through a series of trials and errors), our results strongly suggest that, when weakened hosts are involved, the colonization behaviour of facultative tree-killing bark beetles does not fit the widely accepted paradigm of ‘mass-attack against tree defence’, but rather corresponds to the expected outcome of group foraging for scarce resources with mass-colonization only being an unnecessary consequence of mass-foraging, with costs and benefits that vary widely from those associated with the mass-killing of HTs.

## Supplementary Material

Complementary analysis and simulations

## Data accessibility

Our data are deposited at Dryad Digital Repository: https://doi.org/10.5061/dryad.5q7k4 [61].
