# Supplementary material for: Colonization of weakened trees by mass-attacking bark beetles: no penalty for pioneers, scattered initial distributions and final regular patterns
Source: R Soc Open Sci. 2018 Jan 3;5(1):170454. doi: 10.1098/rsos.170454 (PMC5792868; doi:10.1098/rsos.170454)
Supplement: Complementary analysis and simulations [file rsos170454supp1.pdf]

ROYAL SOCIETY
OPEN SCIENCE

Royal. Society Open Science
doi: 10.1098/rsos.170454

Colonization of weakened trees by mass-attacking bark beetles: no penalty for pioneers, scattered initial distributions and final regular patterns

Supporting Information

Etienne Toffin[a*], Edith Gabriel[b,c], Marceau Louis[d], Jean-Louis Deneubourg[a] and Jean-Claude Grégoire[d]

[a] *Chimie Physique et Biologie Théorique, Université libre de Bruxelles, CP 231, boulevard du Triomphe, B-1050 Bruxelles, Belgium*
[b] *LMA EA2151, Université d'Avignon, F-84000 Avignon, France*
[c] *INRA - Unité BioSP, F-84000 Avignon, France*
[d] *Spatial Epidemiology Lab (SpELL), Université libre de Bruxelles, CP 160/12, 50 av. FD Roosevelt, B-1050 Bruxelles, Belgium*

Keywords: collective foraging, resource partitioning, competition, bark beetles, public information, aggregation
Author for correspondence: Etienne Toffin, e-mail: etoffin@ulb.ac.be

Supplementary information	Justification	Measurements & calculations
S1. Impact of tree age on the colonization pattern	The experimental setup, with trees felled at different times, did not bias the results	Linear regressions:<br>• Age and attacks: *age vs.* H_{END}; *age vs.* λ_{END}<br>• Age and pheromones: *age vs.* t_{pherom}; *age vs.* λ_{pherom}<br>• Age and pattern: *age vs.* $\lambda_{detection}$<br>→No statistically significant relationship, indicating that windthrow age did not influence the observations
S2. Setup of the main experiment	Display the experimental setup	Comments on the figure
S3. Influence of the pheromone dispensers on the spatial patterns	Show that the pheromone dispensers did not influence the spatial patterns	Deviation test:<br>The statistic is defined by the integral deviation measure:<br>$u_{obs} = \int_0^{r_{max}} \left(T_{obs}(r) - \overline{T(r)}\right)^2 dr$<br>→The p-values estimated for this test for the basal and upper segments were 0.355 and 0.366, respectively, showing that the location of the pheromone

		dispenser does not influence entrance hole location.
S4. Validation of the segment experiments. Results of a side study of the spatio-temporal patterns of colonization on whole trees without pheromone dispensers	Confirm the validity of the main experimental setup	Characteristic values of settlement dynamics, spread of points and spatial pattern
S5. Impact of bark texture on entrance hole location	Display the two different bark textures	Comments on the figure
S6. Relationship between plateau values	Characterize both plateaus and their relationship	• Histogram of the proportion of entrance holes occurring during the second stage of colonization (H_{SECOND}/H_{FIRST})<br>• Linear relationship between the number of entrance holes at the first (H_{FIRST}) and second stages (H_{SECOND})<br>→Value of 2nd plateau cannot be predicted from the 1st plateau.
S7. Impact of weather on male landing dynamics	Describe and quantify the impact of weather on male landing	Landing rate at each time step is considered proportional to the number of available beetles and the temperature:<br>$$\frac{\Delta Li}{M - L(i-1)} = \alpha(T)$$<br>→There was a linear relationship between the average temperature (T_{avg}) and the daily fraction of the still available beetles that had landed (ΔL_{avail}).
S8. Relationship between the number of entrance holes, segment area and convex hull area	Understand the linear relationship between A_{hull}, $A_{segment}$ and H_{END}	Simulations of random infestation of segments of varying areas with the SSI (simple sequential inhibition) algorithm.<br>→Area of the convex hull (A_{hull}) increases quickly with the number of holes and tends to reach that of the segment ($A_{segment}$). The proportion of segment area containing entrance holes ($A_{hull}/A_{segment}$) is dependent on the total number of holes and shows the same profile independent of the segment area ($A_{segment}$).
S9. Characterizing the homogeneity of flying insects and the relative susceptibility of each segment	Display the relative susceptibility of each segment	Linear regression of segment densities, Λ, between paired plots at each monitoring step for each paired segment (i, j).<br>→Linear relationships were found for each pair regardless of infestation stage, indicating that the amount of flying insects around each segment was similar. The different regression slope values quantified the intrinsic susceptibility of each segment.

S10. CSR test (complete spatial randomness)	Summarize the CSR test values	Table indicating the values of the *G'(r)* and *L(r)* tests of CSR<br>→9/13 segments exhibited a regular pattern of entrance holes at the end of the observations while the 4 other segments exhibited CSR (random distribution of holes over the segment).
S11. Comparison between the patterns of final hull density (λ_{END}) and the density at which a regular pattern is detected ($\lambda_{detection}$)	Summarize the statistical test	Table indicates the results of a Kruskal-Wallis multiple comparison post-hoc test between characteristic densities: detection of regular pattern ($\lambda_{detection}$), final regular ($\lambda_{regular}$), final random (λ_{random})<br>→Final densities of random and regular patterns were statistically different.
S12. Pattern comparison	Test the repulsion of additional entrance holes by pre-existing ones	Point process pattern independence test between point patterns at times >*t* and times ≤*t*<br>→Half of replicates indicate that new entrance holes fill the empty spaces during infestation.
S13. Spacing *versus* attack density	Test whether inhibition distance remained constant throughout the entire infestation	Point process pattern rescaling test computes the scaling between the observed pattern and the SSI-simulated pattern to determine if the interaction between points is dependent or not on attack density.<br>→Analysis indicated that hole density was the main factor controlling the nearest-neighbor distance and that the experimental attack pattern behaved like an SSI process.
S14. Location of entrance holes over the available length throughout the observations	Display and summarize the statistical test of attack spread along segments throughout the infestation	→There was a linear relationship between the spreads of entrance holes along the X-axis at the first ($spread_1$) and last ($spread_{END}$) counts.<br>→There was no significant relationship between observation time and entrance hole location along the X-axis.
S15. Comparison between the basal and upper segments	Summarize the statistical test	Characteristic values of settlement dynamics, spread of points and spatial pattern for each segment size compared with a Mann-Whitney rank sum test<br>→There were no statistical differences between the basal and upper segments.

S16. Regulation of the colonization dynamics by the inhibition distance	Theoretical exploration of the control of segment saturation by the inhibition distance	Random attacks were simulated with an SSI algorithm on 13 segments with different susceptibilities. Different inhibition distances were simulated. Simulation stopped when it was no longer possible to add new entrance holes (=termination regulated by inhibition distance).<br>→Termination of the attacks (plateau) during our observations cannot be explained by the inhibition distance (MAD=2.5 cm).<br>→Infestation under the rule of a 2.5-cm inhibition distance can lead to densities close to those measured in the literature on standing trees (ca 4 holes/dm²).
S17. Influence of attack age on establishment behaviour	Test the possible impact of a delay between successive attacks and the inhibition distance around pre-existing entrance holes	Linear regression:<br>distance to the closest neighbour *versus* the time difference between the two attacks involved.<br>→The very weak linear relationship indicates that inhibition distance is not influenced by the age of pre-existing neighbouring holes.

When the normality of the distributions was confirmed (Shapiro-Wilk test), the results were presented as mean±sd (*N*); otherwise, medians [Q1; Q3] (*N*) were used. The significance level for all statistical tests was a=0.05.

S1. Impact of tree age on the colonization pattern

<u>All segments</u>

• Age (*age*) *vs.* total number of entrance holes at the end (H_{END})

$H_{END}=-0.08\times age+172.4$, $F_{1,11}=0.79$, P=0.39, $R^2=0.02$

• Age (*age*) *vs.* density of holes at the end (λ_{END})

$\lambda_{END}=-4.2\times10^{-6}\times age+6.8\times10^{-3}$, $F_{1,11}=1.66$, P=0.22, $R^2=0.05$

• Age (*age*) *vs.* pattern

Random: $age=221.3\pm213.1$ days (n=4); regular: $age=288.0\pm169.0$ days (n=9)

Wilcoxon rank sum test: $W=13.5$, P = 0.52

• Age (*age*) *vs.* time of pheromone removal (t_{pherom})

$t_{pherom}=-0.30\times age+296.0$, $F_{1,11}=0.42$, P=0.53, $R^2=0.05$

• Age (*age*) *vs.* density at pheromone removal (λ_{pherom})

$\lambda_{pherom}=-0.2\times10^{-3}\times age+0.337$, $F_{1,11}=3.01$, P=0.11, $R^2=0.14$

<u>Experiments with the infestation pattern characterized as regular</u>

• Age *vs.* density at the detection of a regular pattern ($\lambda_{detection}$)

$\lambda_{detection}=-0.058\times10^{-3}\times age+0.64$, $F_{1,7}=0.03$, P=0.86, $R^2=0.14$

S2. Setup of the main experiment

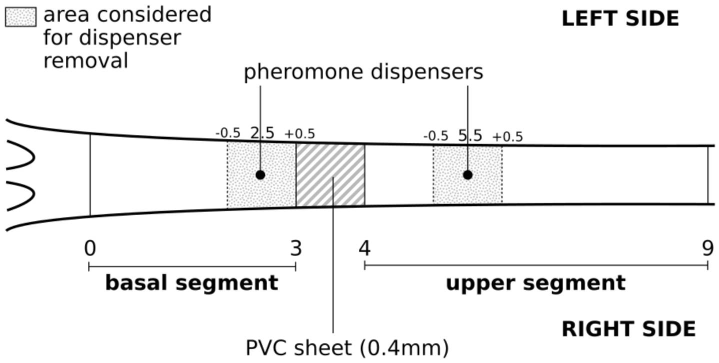

Figure S1. Sketch of a tree (top view). Each tree is separated into 2 observation segments of different sizes (basal and upper segment; distances indicated in metres) by a 1-metre-long PVC sheet. Pheromone dispensers were removed when 20 entrance holes were counted anywhere around the trunks within 50 cm on both sides of the dispensers (area marked by grey shading).

S3. Influence of the pheromone dispensers on the spatial patterns

The potential influence of the pheromone dispensers on the locations of the entrance holes was tested by a deviation test, in which the statistic is defined by the integral deviation measure

$$u_{obs} = \int_0^{r_{max}} \left(T_{obs}(r) - \overline{T(r)}\right)^2 dr \qquad \text{(S1)}$$

where T computes the number of entrance holes within a disc of radius r centred on the pheromone dispenser p, i.e., $T(r) = e_r \#\{x_i \in b(p,r)\}$, and uses *Ripley's* isotropic *edge* correction weights, e_r, to correct for edge effects. In (eq. S1), r_{max} denotes the maximum permissible distance to the pheromone dispenser, T_{obs}. The function T is evaluated from the cumulative entrance hole locations at the time of dispenser removal, and $\bar{T}$ is the mean of $T_1, \ldots, T_N$ obtained from N=999 simulated homogeneous Poisson point processes with intensity related to the number of data locations within the convex hull encompassing the holes. The deviation measure (eq. S1) has been evaluated for each simulated Poisson pattern, and we obtained values $u_1, \ldots, u_N$. The p-value of the deviation test can be estimated as

$$p = 1 - \frac{1}{N+1} \# \{u_j < u_{obs};\ j = 1, \ldots, N\} \qquad \text{(S2)}$$

The p-values for the basal and upper segment were 0.355 and 0.366, respectively. This shows that the location of the pheromone dispenser does not influence entrance hole locations. Complementary analysis also suggests that the pheromone dispensers had no effect on the dynamics of the attacks (see Appendix S12).

S4. Validation of the segment experiments. Results of a side study of the spatio-temporal patterns of colonization on whole trees without pheromone dispensers

		Tree		
		**1**	**2**	**3**
Total number of holes		559	194	188
Density (holes/dm^2)	λ_{END}	0.38	0.34	0.61
Dynamics		$H(t)=559*(1-e^{-0.097t})$; $r^2=0.97$	$H(t)=194*(1-e^{-0.058t})$; $r^2=0.98$	$H(t)=188*(1-e^{-0.039t})$; $r^2=0.97$
Spread	$spread_1$ (cm)	847.1 (0.40 $spread_{END}$); $n=4$	131.55 (0.11 $spread_{END}$); $n=10$	434.5 (0.56 $spread_{END)}$; $n=4$
	$spread_2$ (cm)	1456.6 (0.69 $spread_{END}$); $n=75$	751.6 (0.63 $spread_{END)}$; $n=24$	557.1 (0.72 $spread_{END}$); $n=8$
	$spread_{END}$ (cm)	2118.2; $n=559$	1194.7; $n=194$	769.4; $n=188$
Spread of the holes along the X-axis through time $Spread_X=f(day)$	*slope* (cm/day)	-11.7	-13.6	-12.1
	intercept (cm)	1309.3	918.8	795.9
	R^2 (p-value)	0.03 (<0.0001)	0.18 (<0.0001)	0.39 (<0.0001)
Mean nearest-neighbour distance (cm)	$nnDist_1$	200.5	25.6	121.2
	$nnDist_{END}$	8.2	8.6	7.8
Global pattern		CSR	CSR	regular ($\lambda_{detection}=0.59$)

Table S1. Statistical results of the analysis of whole trees.

S5. Impact of bark texture on entrance hole location

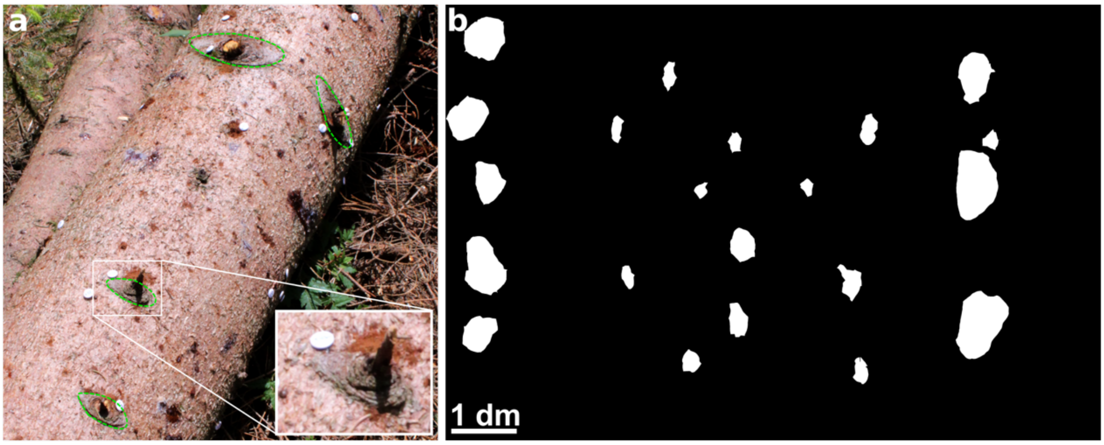

Figure S2. Characterization of bark texture. **a.** Most of the segments are covered by smooth bark, while rough bark, which is delineated with green dashed lines, surrounds the branches (the bottom insert magnifies a rough bark portion). Several entrance holes and their associated numbered white pins are visible. **b.** A portion of segment 14 showing both bark textures (black: smooth; white: rough).

S6. Relationship between plateau values

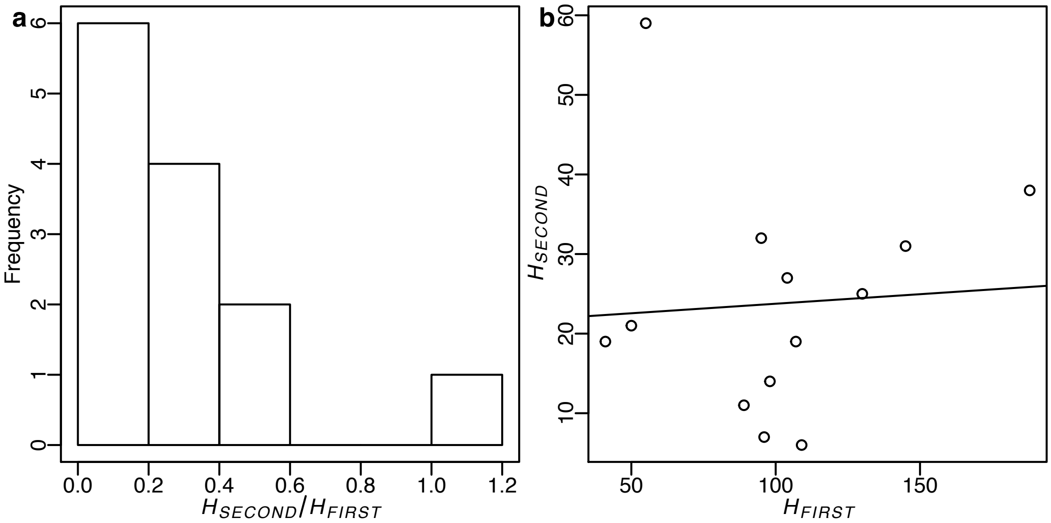

Figure S3. Variation in entrance hole number through the two successive colonization stages. **a.** The proportion of entrance holes occurring during the second stage of colonization (H_{SECOND}/H_{FIRST}) indicates that most holes appeared during the first stage. **b.** The number of entrance holes occurring during the second stage (H_{SECOND}) is not related to the number of holes that appeared during the first stage (H_{FIRST}) (H_{SECOND}=0.02×H_{FIRST}+21.36; $F_{1,11}$=0.05, P=0.83, R^2=0.0).

S7. Impact of weather on male landing dynamics

Weather data (daily average (T_{avg}), minimum (T_{min}) and maximum (T_{max}) temperature) were obtained from the Belgian Royal Meteorological Institute weather station at Beauraing. The impact of temperature on the colonization dynamics was assessed for the overall colonization process by characterizing the relationship between the number of beetles landed on the 4 traps and the 3 corresponding daily temperature values (T_{max}, T_{avg} and T_{min}) (Figure S4 **a**).

The total number of landed insects (L_T) is proportional to the size of the global beetle reservoir, so the number of landings on the traps at each time step is proportional to the number of beetles available during the period:

$$\Delta L(i) = \alpha(T)(L_T - L_{i-1})$$

where i is the day under consideration; ΔL is the number of beetles landed on the trap at day I; T is the temperature; a is the landing rate; and L_{i-1} is the total number of insects landing at day i-1. This equation can also be written as

$$\frac{\Delta Li}{M - L(i-1)} = \alpha(T)$$

where $\frac{\Delta Li}{M-L(i-1)}$ stands for the daily fraction of the still available beetles that have landed (ΔL_{avail}).

There is a linear relationship between the average temperature (T_{avg}) and the ΔL_{avail}, whose slopes correspond to the relationship between the landing rate a=0.036/°C at T_{avg}=17.7±3.2°C (N=16) (Figure S4 **b**).

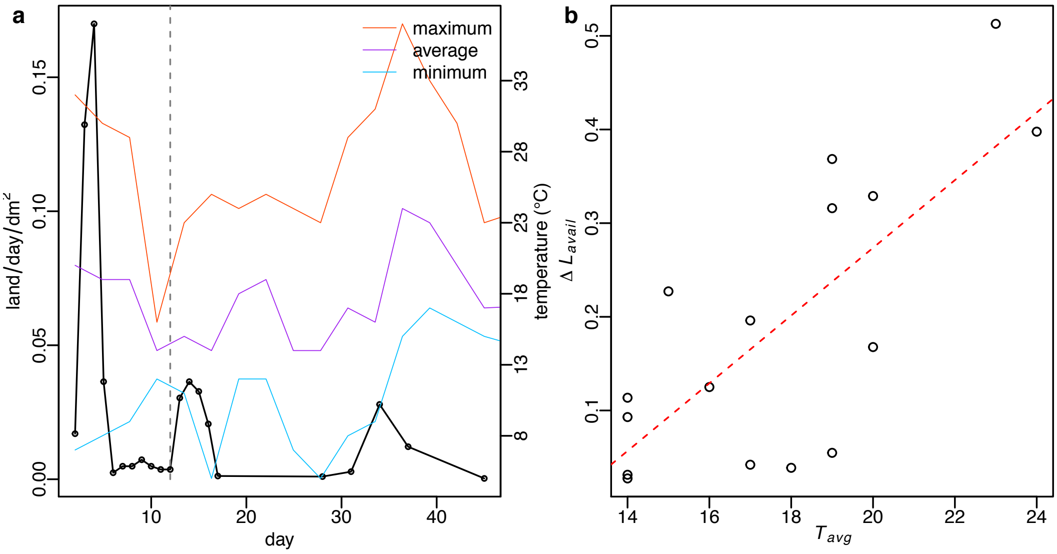

Figure S4. The landing dynamics over the 4 landing traps is strongly related to temperature. **a** Number of beetles captured daily and the characteristic daily temperatures (minimum, average, maximum). **b** Linear relationship between the average temperature (T_{avg}) and the daily fraction of still-available beetles that have landed (ΔL_{avail}) (ΔL_{avail}=0.036 T_{avg} – 0.449; R^2=0.53, $F_{1,14}$=17.6, P=0.001).

S8. Relationship between the number of entrance holes, segment area and convex hull area

We used the SSI algorithm (see Methods – Statistical analysis) to simulate the random infestation of segments under inhibition constraint (MAD=2.5 cm). Simulated segments had a *width*=80 cm under all conditions while *length* varied between 50 and 400 cm to explore the effect of the available surface ($A_{segment}$=*width*×*length*). Thirty replicates were run for each value of $A_{segment}$.
Colonization occurred until saturation of the segment (*giveup*=segment area/4) regardless of the $A_{segment}$ value. Hole density reached values of at least 6 holes/dm^2.

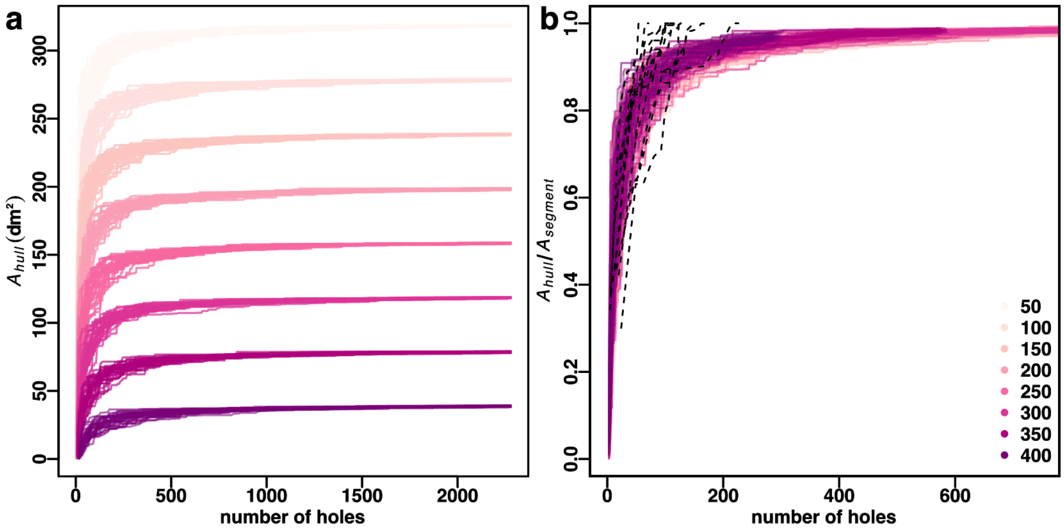

Figure S5. a Under all conditions, the area of the convex hull increases quickly with the number of entrance holes and tends to reach that of the segment. **b** The curves for all conditions overlap well when considering the "convex hull area / segment area" ratio ($A_{hull}/A_{segment}$) relative to the total number of holes. This indicates that, with a similar number of entrance holes and regardless of the available space, the random colonization over the segment area covers the same fraction of the available space. Moreover, when the number of remaining holes is <100, it can predict the $A_{hull}/A_{segment}$ ratio, i.e., the proportion of the segment area covered by the convex hull of entrance holes, well. For each experimental segment, black dashed lines show the fraction of the final convex hull area covered as a function of the number of holes.

S9. Characterizing the homogeneity of flying insects and the relative susceptibility of each segment

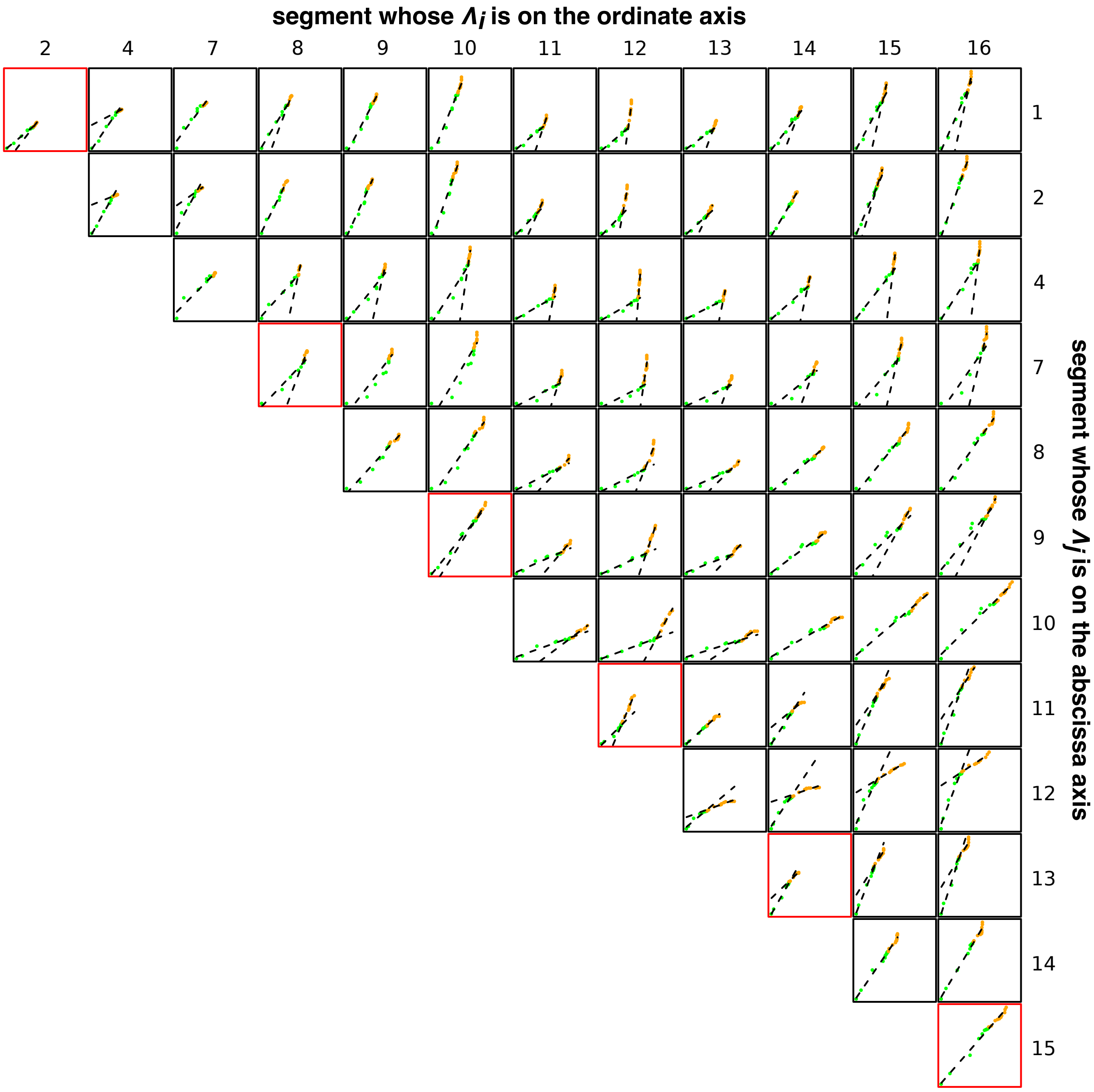

Figure S6. Linear regressions of segment densities, Λ, at each monitoring step for each pair of segments (i, j). Each point in the graphic corresponds to the pair of values, Λ_i and Λ_j, measured at a given monitoring step. Even number correspond to basal segments, while even number stand for upper segments. Red frames highlight relationships between segments belonging to the same tree. Green and orange dots indicate the first and second colonization stages, respectively. Dashed lines represent the linear regressions at each stage.

S10. CSR Test

		final number of holes (H_{END})	final hull density (λ_{END}; holes/dm^2)	G'-based dclf test p-value	L-based dclf test p-value
segment	**1**	100	0.524	**0.005**	**0.002**
	2	125	0.409	**0.035**	0.109
	~~3~~	~~15~~	~~-~~	~~-~~	~~-~~
	**4**	115	0.718	**0.036**	**0.001**
	~~5~~	~~30~~	~~-~~	~~-~~	~~-~~
	~~6~~	~~19~~	~~-~~	~~-~~	~~-~~
	**7**	103	1.122	**0.002**	**0.001**
	**8**	154	0.844	**0.009**	**0.001**
	**9**	112	0.748	**0.014**	**0.001**
	**10**	176	0.762	**0.021**	**0.003**
	11	71	0.570	0.196	**0.004**
	**12**	114	0.554	**0.011**	**0.001**
	13	60	0.454	0.226	0.105
	14	127	0.582	0.073	**0.001**
	**15**	131	0.730	**0.027**	**0.002**
	**16**	226	0.917	**0.002**	**0.001**
tree	1	559	0.376	**0.016**	0.234
	2	194	0.337	0.490	0.166
	3	188	0.614	0.062	**0.003**

Table S2. Statistical results from the analysis of segments and whole trees. Strikethrough values indicates the segments discarded because of apparent bark decay (which may explain their low number of entrance holes).

S11. Comparison between the patterns of final hull density (λ_{END}) and the density at which a regular pattern is detected ($\lambda_{detection}$)

	**Observed difference**	**Critical difference**	**Statistical difference**
λ_{random} - $\lambda_{detection}$	54.167	93.417	FALSE
λ_{random} - $\lambda_{regular}$	98.611	93.417	TRUE
$\lambda_{detection}$ - $\lambda_{regular}$	44.444	73.282	FALSE

Table S3. Statistical results from a Kruskal-Wallis multiple comparison post-hoc test (Kruskal-Wallis $\chi^2_2 = \mathbf{6.637}$, P=0.036). $\lambda_{regular}$: final hull densities with regular patterns; λ_{random}: final hull densities with random patterns; $\lambda_{detection}$: hull densities whose patterns are detected as regular.

S12. Pattern comparison

To test for independence between point patterns at times $>t$ and times $\leq t$, we adapted Lotwick and Silverman's [1] and Van Lieshout and Baddeley's [2] approaches for classifying the spatial pattern of points into distinct types. These authors test the null hypothesis that the sub-patterns of the points of each type are independent point processes by wrapping the study window into a torus, fixing locations of type-1, shifting locations of type-2 on the torus and comparing the nearest-neighbour distributions. In our case, type-1 events (resp. type-2 events) correspond to locations at times $>t$ (resp. times $<t$).
For half of the replicates, we concluded that there is a repulsion between the two point patterns, meaning that the points do interact and that new entrance holes fill the empty spaces. These results were obtained when comparing the attack patterns before and after any arbitrary time t (including before the removal of pheromones, which also strongly suggests that the pheromone dispensers did not influence the location of the entrance holes). For the remaining replicates, non-significance may have been linked to the small number of points used to test for independence.

S13. Spacing *versus* attack density

In our observations, the nearest-neighbour distance between points decreases as the number of points increases. A relevant question is whether the interaction strength (e.g., inhibition distance) between points remained constant over time independent of hole density.
We considered the point pattern at time t to be a scaled version of the pattern at time $t+1$ [3]. Thus, for each point pattern X_t at each time t, we computed a scale factor c_t such that the intensity of X_t/c_t is equal to one. Then, we computed the mean nearest-neighbour distance between the points of X_t/c_t and X_{t+1}/c_t, which should tend toward a constant if the interaction between points does not depend on attack density.
We computed the nearest-neighbour distances obtained from the patterns of attacks and from 99 regular processes simulated with an SSI algorithm (see Methods – Statistical analysis) with the same densities. Figure S7 compares the nearest-neighbour distances of the rescaled patterns, and it clearly suggests that entrance hole density is the main factor controlling nearest-neighbour distance and that, from this point of view, the experimental attack pattern behaves like an SSI process.

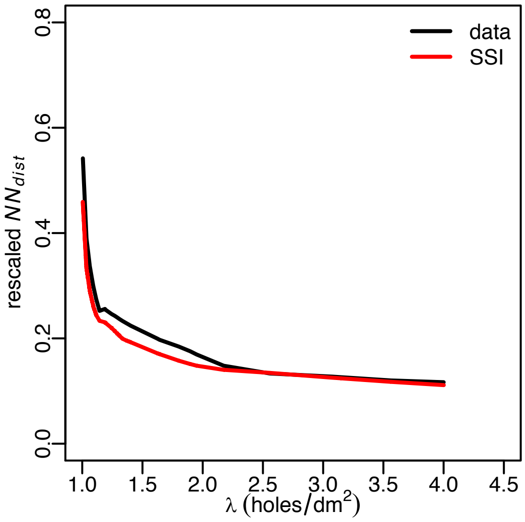

Figure S7. Nearest-neighbour distance between patterns at times *t* and *t*+1 after rescaling.

S14. Location of entrance holes over the available length throughout the observations

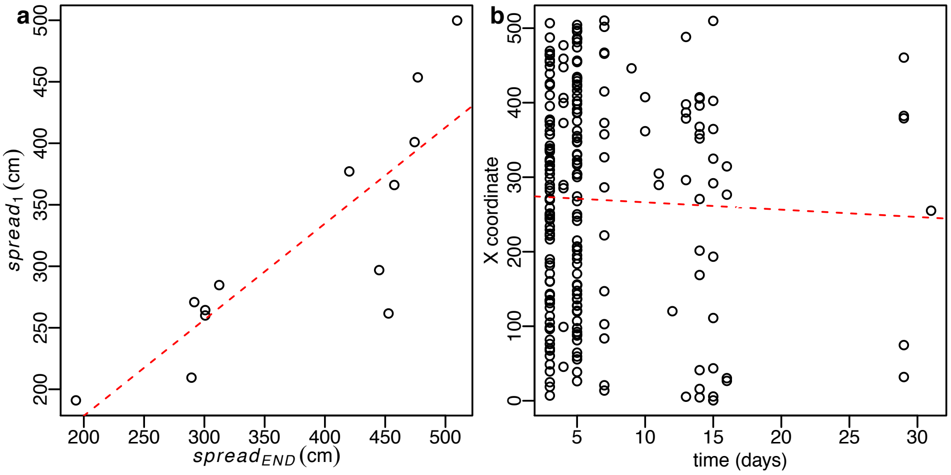

Figure S8. a The spreads of entrance holes along the X-axis at the first (*spread₁*) and last (*spread*$_{END}$) counts show a strong linear relationship ($spread_1$=0.782×$spread_{END}$+22.120, $F_{1,11}$=25.5, P<<0.001, R^2=0.67). **b** Entrance holes are randomly scattered over the whole segments throughout the observations. Replicate 16, which is represented here, as well as the 8 other replicates, does not show any significant relationship between observation time and entrance hole location along the X-axis (linear regression parameters in Table S3).

						X=f(day)			
replicate	n_1	n_{END}	***time*** $H=0.9H_{END}$ ***(days)***	***range*** X_1 ***(cm)***	***range*** X_{END} ***(cm)***	***intercept (cm)***	***slope (cm/day)***	***p-value***	R^2
1	23	100	14	271	292	145.3	-0.014	0.84	0
2	24	126	15	297	445	**114.3**	**0.424**	**<0.001**	**0.20**
4	21	115	7	377	420	118.5	0.104	0.30	0.01
7	49	103	6	191	193	218.1	0.044	0.52	0
8	39	155	14	366	458	**112.2**	**0.555**	**<0.001**	**0.21**
9	13	112	15	260	301	142.7	0.067	0.33	0.01
10	16	176	28	454	477	244.9	-0.041	0.63	0
11	7	71	34	264	301	138.4	0.052	0.36	0.01
12	5	114	34	401	474	203.9	0.043	0.52	0
13	6	60	17	209	289	138.7	0.099	0.14	0.04
14	12	127	15	262	453	**269.8**	**-0.251**	**0.001**	**0.08**
15	20	131	28	285	312	**180.8**	**-0.154**	**0.01**	**0.05**
16	36	226	34	500	510	275.8	-0.042	0.58	0

Table S4. Variation in the timing of entrance hole location along the X-axis.

S15. Comparison between the basal and upper segments

		Basal (N=6)	Upper (N=7)	W	p-value
**Establishment dynamics**	β (day^{-1})	0.15 [0.13;0.2]	0.17 [0.13;0.19]	21	1
**Pheromone removal**	time (h)	149 [96;344]	98 [79;102]	27.5	0.39
	λ_{pherom} (holes/dm^2)	0.50 [0.43;0.60]	0.47 [0.39;0.50]	29	0.29
	fraction of total number of holes (H_{pherom}/H_{END})	0.80 [0.56;0.91]	0.57 [0.49;0.62]	30	0.23
**Final observation**	λ_{END} (holes/dm^2)	0.65 [0.54;0.74]	0.72 [0.57;0.80]	19	0.84
**Spatial pattern**	$spread_1/seg.length$	0.87 [0.74;0.90]	0.75 [0.66;0.86]	25	0.63
	$spread_{END}/seg.length$	0.99 [0.97;1.00]	0.92 [0.90;0.95]	31	0.18
	$nnDist$ (cm)	7.8 [6.9;8.6]	7.1 [6.5;8.5]	26	0.53
	$\lambda_{detection}$ (holes/dm^2)	0.68 [0.63;0.74]	0.70 [0.56;0.71]	10	1

Table S5. Results of Mann-Whitney statistical comparison of spatio-temporal colonization of the segments depending on their length (basal = 3 m; upper = 5 m).

There are no statistical differences between the basal and upper segments in terms of both the establishment dynamics (colonization rate β) and the density at the final observation (λ_{END}), indicating that the available space at the investigated ranges did not influence colonization intensity. The comparison of the different variables characterizing pheromone removal (time, λ_{pherom}, and the fraction of the total number of entrance holes at H_{END}) also supports this result. Similarly, the spatial pattern on both segment types did not show any difference (spread over time, nearest-neighbour distance between holes, and the density at which the pattern was characterized as regular).

S16. Regulation of the colonization dynamics by the inhibition distance

The effect of the inhibition distance on the colonization dynamics was tested by simulating random attacks until the available surface was saturated.

Random attacks were simulated within an environment consisting of 13 segments of area A_i and susceptibility a_i using an SSI algorithm (see Methods) with various inhibition distances, MAD (cm). In this simulation, the location of the new entrance holes was determined using weighted random sampling (weighted by segment susceptibility a_i). The simulation terminated when the number of entrance holes stopped increasing for *giveup*=10,000 successive iterations (maximal number), suggesting that it was no longer possible to add new entrance holes. Hence, the infestation dynamics and termination were only regulated by the inhibition distance. The total number of entrance holes at the end of the simulation is H.

The area and susceptibility of each segment were similar to those of an experimental segment; the MAD value ranged from 0 cm to 25 cm (MAD=0 cm indicates no inhibition; in this case, the simulation terminates when the total number of entrance holes reaches N=8,000 holes). Each segment i bears a number of holes H_i defined as

$$H_i = \frac{\alpha_i A_i}{\sum_{i=1}^{13} \alpha_i A_i} H$$

and has an infestation density of λ_i

$$\lambda_i = \frac{\alpha_i}{\sum_{i=1}^{13} \alpha_i A_i} H$$

Thus, there is a theoretical linear relationship between the susceptibility a_i, and the density of the segments with a slope $\frac{H}{\sum_{i=1}^{13} \alpha_i A_i}$.

At the end of our simulations, this linear relationship between susceptibility and density was verified throughout the entire colonization process when MAD=0 cm (no inhibition, Figure S9 a & b). In contrast, for MAD≥2.5 cm, the density tended to reach a plateau on the most receptive segments while continuing to increase on the others. This is particularly visible for large values (MAD≥15 cm), at which the final hole density was similar on each segment, but this was not compatible with our observed results of MAD≈2.5 cm.

Moreover, at MAD values closer to our observed results (MAD≤9 cm), the total number of entrance holes at the end of the simulation was far greater than the total number recorded on any segment throughout the observations was H_{END}=1614 holes (Table S6, Figure S9 a). Hence, when considering only the first 1614 entrance holes of our simulations, the relationship between segment susceptibility and density remained linear for MAD≤9 cm (Figure S9 b). This indicates that the termination of the attacks (plateau) that occurred during our observations cannot be explained by the inhibition distance (MAD=2.5 cm).

Our simulations also show that infestation under the rule of a 2.5 cm inhibition distance can lead to densities close to those measured in the literature on standing trees (ca 4 holes/dm^2) (Table S6 and see Discussion).

MAD (cm)	*N* (holes)	λ (holes/dm^2; min-max)
0.0	8000	1.06–3.67
2.5	7679	1.11–3.13
3.5	6376	1.07–2.39
4.5	5114	0.98–1.87
5.5	4224	0.88–1.44
6.5	3425	0.74–1.15
9.0	2248	0.52–0.71
15.0	998	0.24–0.31
25.0	419	0.10–0.13

Table S6. Total number of entrance holes and density range at saturation for each MAD value.

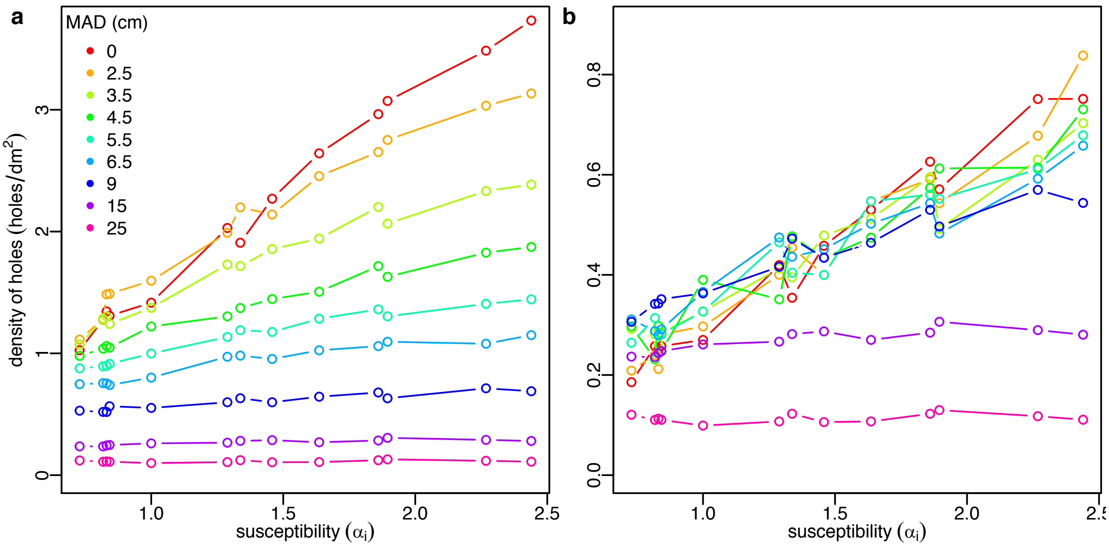

Figure S9. Variation in entrance hole density on the simulated segments as a function of segment susceptibility, at **a,** the end of the simulation, and **b,** when the highest observed final number of entrance holes, *N*=1614, is reached. Each point stands for one of the 13 simulated segments.

S17. Influence of attack age on establishment behaviour

The pheromones emitted by male *Ips typographus* vary with the size of the galleries, i.e., with the time since gallery initiation [4]. Therefore, the question of whether galleries of different ages could have different spatial influences on further settlement arose. To explore this issue, we computed the distance to the closest neighbour and the time difference between two attacks. The results show a statistically significant but weak linear relationship (R^2=0.02, Figure S10) between the age of an attack and the distance to its closest neighbour.

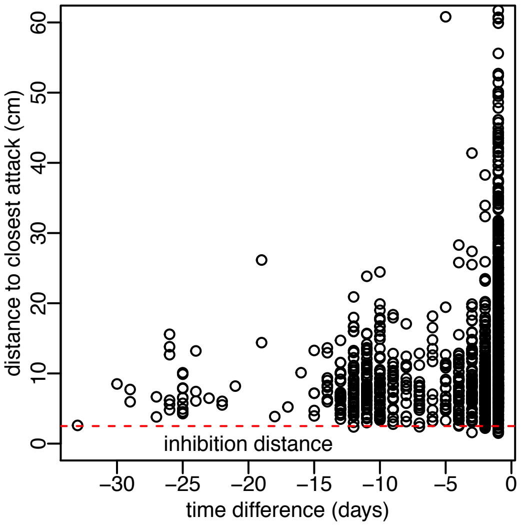

Figure S10. Distance to the closest neighbour *versus* the time difference between two attacks. The linear relationship is statistically significant but very weak (*distance_closest_attack*=-0.80×*time_difference*+15.63, $F_{1,1341}$=31, P<<0.001, R^2=0.02).

References

1. Lotwick HW, Silverman BW. 1982 Methods for Analysing Spatial Processes of Several Types of Points. *J. R. Stat. Soc. Ser. B* **44**, 406–413.
2. Van Lieshout MNM, Baddeley AJ. 1999 Indices of Dependence Between Types in Multivariate Point Patterns. *Scand. J. Stat.* **26**, 511–532. (doi:10.1111/1467-9469.00165)
3. Hahn U, Vedel Jensen EB, Van Lieshout MC, Nielsen LS. 2003 Inhomogeneous spatial point processes by location-dependent scaling. *Adv. Appl. Probab.* **35**, 319–336. (doi:10.1239/aap/1051201648)
4. Birgersson G, Schlyter F, Löfqvist J, Bergström G. 1984 Quantitative variation of pheromone components in the spruce bark beetle *Ips typographus* from different attack phases. *J. Chem. Ecol.* **10**, 1029–1055. (doi:10.1007/BF00987511)
